# Comparison of different preoperative fasting times for pediatric elective surgical procedures: A randomized controlled trial

**DOI:** 10.12669/pjms.40.7.8403

**Published:** 2024-08

**Authors:** Humna Jasarat, Fatima Naumeri

**Affiliations:** 1 Humna Jasarat, MBBS. Department of Pediatric Surgery, King Edward Medical University/Mayo Hospital, Lahore, Pakistan; 2 Fatima Naumeri, MBBS, MCPS, FCPS, MCPS-HPE, CME. Department of Pediatric Surgery, Services Institute of Medical Sciences/ Services Hospital, Lahore, Pakistan

**Keywords:** Standard fasting, Liberal fasting, General Anesthesia, Surgical Procedures, Nil Per Oral

## Abstract

**Objective:**

To compare the mean residual gastric volume and gastric pH with standard and liberal fasting in children undergoing general anesthesia for elective procedures.

**Methods:**

A randomized controlled trial (NCT 05922072) was conducted at Department of Pediatric Surgery, Mayo Hospital Lahore from June 2021 to December 2021 and 120 patients undergoing elective daycare surgical procedures under general anesthesia were enrolled. Patients were divided into Group-A (Standard fasting) and Group-B (Liberal fasting). Group-A with 6-, 4- and 2-hours Nil per oral (NPO) for solids/ formula milk, breast milk and clear fluid, while Group-B with six, four and one hour NPO for solids/ formula milk, breast milk and clear fluid respectively. Residual gastric volume and pH were measured after anesthetizing the patient. Effect modifiers like age, gender, duration of anesthesia and procedure were controlled through stratification. Post-stratification, t-test was applied and p-value less than 0.05 was taken as statistically significant.

**Results:**

In Group-A, mean age was 6.1±4.5 years and 6.4±4.6 years in Group-B. Mean residual gastric volume with standard fasting was 0.67±0.48 ml and liberal fasting 0.80±0.44 ml (p value 0.13). Mean gastric fluid pH with standard fasting was 1.72±0.78 as compared to liberal fasting 1.63±0.70 (p value 0.53).

**Conclusion:**

Free fluid fasting allows for significantly shorter fasting times, though statistically insignificant higher residual gastric volume was recorded in liberal fasting group with a lower pH.

## INTRODUCTION

Pre-operative fasting is a specific time-period before a procedure in which patient is advised not to take any liquid or solid by mouth.^1^ In the period of chloroform anesthesia, Nil per oral (NPO) was introduced because of vomiting and discomfort associated with anesthesia, and to prevent aspiration of gastric contents during general anesthesia (GA). Since then, NPO has been a universally acceptable practice before GA. With introduction of better anesthesia drugs recommendations for minimal fasting time before general anesthesia have changed.^2^,^3^

Recent guidelines suggest six, four and two hours fasting for solids/ infant formula milk, breast milk and clear fluids.^4^ However, many times, children suffer excessive unnecessary fasting due to delay in surgery which may reduce systolic blood pressure, induce catabolic state, and cause behavioral effects.^5^,^6^ Moreover prolonged fasting increases insulin resistance and may increase the inflammatory response to surgery.^7^ Shortening fasting time is theoretically associated with increased risk of pulmonary aspiration. Pulmonary aspiration of gastric contents is a rare event counting for nine out of 10,000 children.^8^ The increased risk may be measured indirectly through Gastric residual volume and pH. Studies suggest that mean residual gastric volume and gastric pH don’t differ significantly whether NPO for clear fluids is of one hour or two hours.^9^ In a recent consensus statement, anesthesiologists recommend taking clear fluids up till one hour before general anesthesia, for children.^10^

A randomized controlled trial was conducted on children in 2018 concluded that two hours standard NPO resulted in longer fasting times than liberal fasting (clear fluids until premedication with midazolam). However, in patients with less than half an hour of liberal fasting, elevated gastric volumes were noted.^11^ The rationale of this study was to provide evidence on minimal safe clear fluid fasting duration without the risk of aspiration of gastric contents during elective pediatric procedures under general anesthesia. In our setting patients suffer longer fasting that causes increased thirst and irritability among children. This study will help in establishing the local guidelines and change practice towards shorter fasting times.

## METHODS

This randomized controlled trial (NCT 05922072) was conducted at Department of Pediatric Surgery, Mayo Hospital, and Lahore from June to December 2021. One hundred twenty patients of both gender between 1 to 12 years of age and undergoing elective daycare surgical procedures (Orchidopexy, Lymph node biopsy, Skin grafting, Tongue tie release/ circumcision or Herniotomy) under general anesthesia were enrolled in the study. Patients with co-morbidities like history of dyspepsia, treatment of reflux or gastritis, family history or self-history of diabetes mellitus, and/or neurologically impaired were excluded. The sample size of 120 (60 in each group) was estimated by 95% confidence interval with 80% power of test and taking an expected mean residual volume as 0.41±0.28 ml with standard fasting^7^ and 0.91±1.32 ml with liberal fasting.^11^

### Ethical Approval:

After taking ethical approval (IRB 492/RC/KEMU, dated: 09/07/2021).

**Fig.1:**
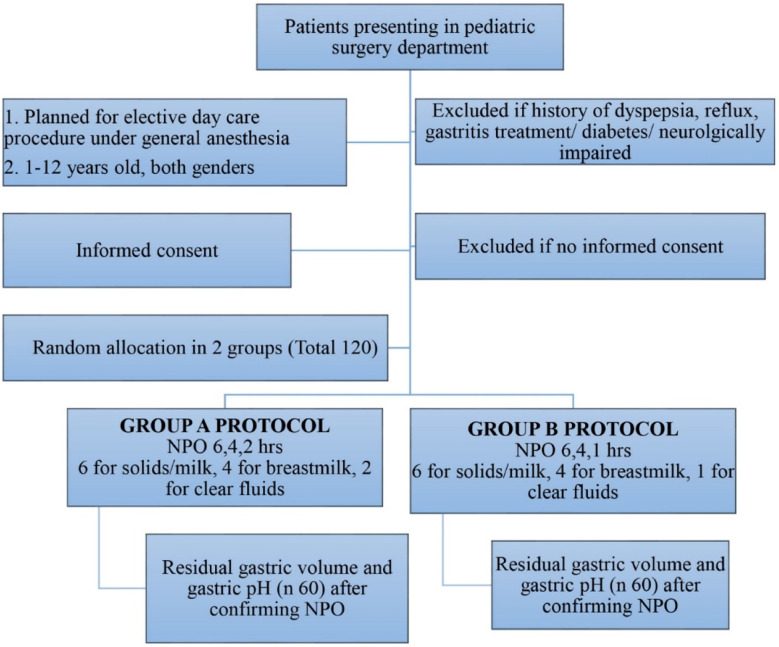
Flow chart of patient selection, allocation, and outcome analysis of trial.

After getting informed consent from parents, patients were randomly allocated into two groups (60 in each group) using computerized generated numbers (Fig.1). Group-A was standard fasting group with six, four and two hours NPO for solids/ formula milk, breast milk and clear fluid respectively, while Group-B was liberal fasting group with six, four and one-hour NPO for solids/ formula milk, breast milk and clear fluid respectively. All patients had preoperative assessment and fit patients were prepared for general anesthesia and selected using purposive sampling. Premedication was given one hour before surgery and NPO protocol was confirmed before elective general anesthesia.

After anesthetizing, a nasogastric tube (age specific formula for size of NG tube = 16+age in years/two) was used to take gastric sampling. Residual gastric volume was measured by using 60 cc syringe. pH was calculated by pH litmus paper strip on gastric aspirate. Pulmonary aspiration was identified by either intraoperative hypoxia on pulse oximeter or presence of bilious secretions or particulate matter in the pulmonary tree by laryngoscopy/ bronchoscopy and post-operatively it was determined by clinical assessment (Fever, cough and conducting sounds on chest auscultation and chest radiograph in symptomatic patients that confirmed infiltrates in basal segments of posterior lobes of lungs).

Data were entered and analyzed by using SPSS version 25.0. Mean and standard deviation was calculated for quantitative variables (age of the patient, duration of surgery, gastric residual volume and gastric pH). Frequency and percentage were calculated for qualitative variable (gender of the patient, types of surgical procedures). Both groups were compared by using t-test. Effect modifiers like age, gender, duration of anesthesia and procedure were controlled through stratification. Post-stratification, t-test was applied and p-value less than 0.05 was considered statistically significant.

## RESULTS

A total of 120 were enrolled and randomly allocated in Group-A (Standard fasting) and Group-B (Liberal fasting). In Group-A, 37(61.7%) patients were male and 23(38.3%) were female. In Group-B, 36(60%) patients were male and 24(40%) were female. In Group-A, the mean age was 6.1±4.5 years and 6.4±4.6 years in Group-B. In Group-A, 25(41.7%) patients were in the 1-6 years age group, while 35(58.3%) were in 7-12 years age group. In Group-B, 23(38.3%) patients were in the 1-6 years age group, while 37(61.7%) were in the 7-12 years age group.

In Group-A, 36(60%) patients had less than one hour duration of anesthesia, while 24(40%) had more than one hour duration. In Group-B, 34(56.7%) patients had less than one hour duration of anesthesia, while 26(43.3%) had over one hour anesthesia. Mean residual gastric volume with standard fasting was 0.67±0.48 ml, as compared to liberal fasting 0.80±0.44 ml. The difference was insignificant (p 0.133). Mean gastric fluid pH with standard fasting was 1.72±0.78, and in liberal fasting group 1.63±0.70. The difference was insignificant (p 0.528) between groups.

No significant difference was found between the residual gastric volume and gastric fluid pH with study groups stratified by age and duration of anesthesia. Gender stratification revealed higher residual volume for girls in liberal fasting group than standard fasting group (p-value 0.04) (Table-I)

**Table-I:** Stratification of outcome between groups with respect to gender, age, duration of anesthesia.

Variables		OUTCOMES					

		Residual gastric volume (ml)			Gastric pH		

		Group-A	Group-B	P value	Group-A	Group-B	P-value
Gender	Male	0.67±0.51	0.71±0.43	0.768	1.81±0.78	1.72±0.74	0.633
	Female	0.68±0.45	0.96±0.43	0.04	1.58±0.76	1.51±0.63	0.710
Age (years)	1-6	0.56±0.47	0.80±0.51	0.098	1.63±0.81	1.67±0.58	0.857
	7-12	0.75±0.49	0.80±0.39	0.639	1.79±0.76	1.62±0.78	0.348
Anesthesia Duration (hours)	< 1	0.65±0.48	0.77±0.46	0.303	1.78±0.75	1.57±0.65	0.216
	≥ 1	0.71±0.49	0.85±0.41	0.293	1.63±0.82	1.72±0.77	0.695

No significant difference was found between the residual gastric volume and gastric fluid pH with study groups stratified by surgical procedures, except gastric fluid pH in orchidopexy (Table-II).

**Table-II:** Stratification of outcome between groups with respect to surgical procedure.

Surgical procedure	Outcome	Groups	(n) Mean ± Std. Deviation	p-value
Orchidopexy	Residual gastric volume (ml)	Group-A	(15) 0.64±0.47	0.720
		Group-B	(14) 0.69±0.42	
	Gastric fluid pH	Group-A	(15) 1.97±0.81	0.013
		Group-B	(14) 1.25±0.64	
Lymph node biopsy	Residual gastric volume (ml)	Group-A	(11) 0.83±0.57	0.841
		Group-B	(10) 0.88±0.53	
	Gastric fluid pH	Group-A	(11) 1.64±0.76	0.942
		Group-B	(10) 1.67±0.65	
Skin grafting	Residual gastric volume (ml)	Group-A	(8) 0.46±0.50	0.108
		Group-B	(12) 0.85±0.62	
	Gastric fluid pH	Group-A	(8) 1.83±0.71	0.678
		Group-B	(12) 1.96±0.47	
Tongue tie release/ circumcision	Residual gastric volume (ml)	Group-A	(12) 0.55±0.45	0.201
		Group-B	(11) 0.81±0.73	
	Gastric fluid pH	Group-A	(12) 1.56±0.79	0.780
		Group-B	(11) 1.65±0.41	
Herniotomy	Residual gastric volume (ml)	Group-A	(14) 0.83±0.36	0.954
		Group-B	(13) 0.82±0.89	
	Gastric fluid pH	Group-A	(14) 1.59±0.63	0.684
		Group-B	(13) 1.71±0.75	

## DISCUSSION

In this study, liberal oral clear fluid intake until one hour before procedure time was compared with the conventionally prescribed two hours of clear fluid fasting for indirectly evaluating gastric contents aspiration, through gastric volume, and it was noted that mean gastric residual volume and pH remained almost similar in both groups, though statistically insignificant high gastric residual volume with low pH was noted in the liberal fasting group.

Prolonged fasting times affect hydration status, cause irritability and stress, and lead to decreased blood pressure.^12^-^15^ Sumpelmann and colleagues recommend shortening fasting times to avoid patient discomfort, dehydration, and ketoacidosis.^16^ Delay in procedures, change in schedules, communication problems mainly lead to prolonged fasting times, even exceeding current recommendations for clear fluid fasting.^6^,^17^-^22^ A local study found that allowing half a liter of oral rehydrating solution two hours prior to anesthesia induction, didn’t cause nausea or mortality in any patient, although they didn’t evaluate gastric fluid volumes.^23^ Dennhardt and colleagues^5^ found that constantly updating fasting orders and encouraging feed till updated time of surgery in children improves metabolic and hemodynamic stability at time of induction. Another study found that shortening fasting times results in shorter hospital stays and is cost effective.

In this study, mean residual gastric volume with standard fasting was (0.67±0.48 ml) as compared to liberal fasting (0.80±0.44 ml), and mean gastric fluid pH with standard fasting was (1.72±0.78) as compared to liberal fasting (1.63±0.70). The difference was insignificant (p>0.05) between groups. In another similar study, the pH was 1.55±0.68 in standard and 1.44±0.26 in liberal group, while mean residual volume was 0.50±0.4 and 0.64±0.63 respectively.^9^ Similar findings were reported by a study where the mean pH and gastric fluid volume in standard group was 1.9±0.5 and 0.41±0.28.^7^

In 2018, a randomized controlled trial was conducted on children in which one group followed two hours standard NPO and the second group was allowed to take clear fluid until premedication with midazolam (liberal fasting). In this study the liberal fasting group had shorter fasting times (106.0±185.86 minutes) than standard fasting group (370.9±248.1 minutes) (p<0.001), while no significant difference was observed regarding mean gastric pH (1.7±0.69 in liberal group vs. 1.6±0.27 in standard group) or residual volume (0.91±1.32 vs. 0.53±0.50ml). However, twelve patients (15%) in the liberal group (median fluid fasting 32 minutes vs. one patient (1%) had gastric residual volumes >2 ml/kg (P<0.001), so recommendation of fasting time over half hour was suggested.^11^

Residual gastric volumes more than 2 ml/kg body weight may be measured with prolonged clear fluid fasting times.^17^,^24^ However, before induction of anesthesia fluid restriction is recommended to avoid perioperative pulmonary aspiration.

This study helped in establishing guidelines in local context, and preventing excessive unnecessary fasting in children. Minimal safe fating time was assessed through residual gastric volume and its pH and can predict the risk of aspiration pneumonia indirectly. In short, this study adds to previous consensus statement of safe fasting.^10^

### Limitations:

It included limited co-operation by the anesthesia team, lack of similar previous local research, and conflicts arising from time constraints due to work overload. Patients were selected using nonprobability sampling due to lack of sampling frame and shorter duration of study which may have led to selection bias.

## CONCLUSION

Free fluid fasting allows for shorter fasting times, though statistically insignificant higher residual gastric volume was recorded in liberal fasting group with a lower pH.

### Authors Contribution:

**HJ:** Did data collection and writing, statistical analysis, and approved manuscript.

**FN:** Designed, edited, approved and is accountable for manuscript.
